# Experiences with a blended cognitive behavioral therapy (bCBT) intervention for the treatment of depression and anxiety in university students: A qualitative study

**DOI:** 10.1192/j.eurpsy.2023.984

**Published:** 2023-07-19

**Authors:** P. Braun, E. Atik, L. Guthardt, J. Apolinário-Hagen, M. Schückes

**Affiliations:** 1 Faculty of Medicine, Centre for Health and Society, Institute of Occupational, Social and Environmental Medicine, Düsseldorf; 2 Faculty of Psychology and the Institute of Philosophy II, Ruhr University Bochum, Bochum; 3 Institute for SME Research and Entrepreneurship, University of Mannheim, Mannheim, Germany

## Abstract

**Introduction:**

Internet-based cognitive behavioral therapy (iCBT) programs have been widely acknowledged as effective resources to treat common mental health disorders (CMDs) like depression or anxiety. However, real-world uptake rates remain low, which could be associated to low individualization options of iCBT. Blended cognitive behavioural therapy (bCBT) allows for more personalized care by combining regular face-to-face therapy sessions with digital therapeutics (DTx). However, in-depth experiences with DTx in bCBT programs have yet rarely been examined. In this study, we focused on university students as they are particularly at risk for developing CMDs.

**Objectives:**

The aim of this study was to evaluate experiences with the smartphone-based DTx *elona therapy* among university students with mild to moderate depression or anxiety symptoms for the use within bCBT.

**Methods:**

Semi-structured interviews were conducted via videoconference between January and April 2022 with N = 102 students from universities in North Rhine-Westphalia, Germany, after they had received weekly individual CBT sessions (25 minutes each) via videoconference for six weeks and regularly used the depression (N = 67) or anxiety module (N = 35) of the DTx. Interviews were coded according to the approach of grounded theory.

**Results:**

In general, most participants stated that they benefitted from the bCBT program. Many highlighted the intuitive handling of the DTx and indicated that they perceived it as useful for structuring their therapy progress. As other benefits, participants listed e.g., increased self-reflection and disorder-specific knowledge as well as the transfer of the content of therapy sessions into their daily life. Participants differed with respect to the preferred design of the DTx. While some liked the clean look, others would have favoured more colours. Participants mentioned time constraints, data security concerns or the feeling of being left alone with potentially arising emotions while working on tasks for the next therapy session as possible barriers to the usage of DTx.

**Conclusions:**

Interviewed participants mostly had positive attitudes toward *elona therapy* as part of the bCBT program. Our study shows that DTx as part of bCBT can be perceived as helpful tools to accompany university students with mild to moderate anxiety or depression symptoms in their daily life.

**Disclosure of Interest:**

P. Braun: None Declared, E. Atik Employee of: Elona Health GmbH, L. Guthardt: None Declared, J. Apolinário-Hagen: None Declared, M. Schückes Shareolder of: Elona Health GmbH

